# Glial Fibrillary Acidic Protein in Blood as a Disease Biomarker of Neuromyelitis Optica Spectrum Disorders

**DOI:** 10.3389/fneur.2022.865730

**Published:** 2022-03-17

**Authors:** Hyunjin Kim, Eun-Jae Lee, Young-Min Lim, Kwang-Kuk Kim

**Affiliations:** ^1^Department of Neurology, Asan Medical Center, University of Ulsan College of Medicine, Seoul, South Korea; ^2^Department of Medicine, Asan Medical Institute of Convergence Science and Technology, Seoul, South Korea

**Keywords:** glial fibrillary acidic protein, neuromyelitis optica spectrum disorder, blood, biomarker, anti-aquaporin-4 antibodies, GFAP, NMOSD

## Abstract

Glial fibrillary acidic protein (GFAP) is a type III intermediate filament protein found in astrocytes in the brain. Damaged astrocytes release GFAP into cerebrospinal fluid and blood. Thus, GFAP levels in these body fluids may reflect the disease state of neuromyelitis optica spectrum disorder (NMOSD), which includes astrocytopathy, characterized by pathogenic antibodies against aquaporin 4 located on astrocytes. Recently, single-molecule array technology that can detect these synaptic proteins in blood, even in the subfemtomolar range, has been developed. Emerging evidence suggests that GFAP protein is a strong biomarker candidate for NMOSD. This mini-review provides basic information about GFAP protein and innovative clinical data that show the potential clinical value of blood GFAP levels as a biomarker for NMOSD.

## Introduction

Neuromyelitis optica spectrum disorder (NMOSD) is a chronic inflammatory disease of the central nervous system (CNS) (1, 2). The main pathogenesis of NMOSD is autoimmune channelopathy/astrocytopathy that targets the water channel aquaporin-4 (AQP4) on perivascular astrocytic endfeet, and antibodies against AQP4 (AQP4-Ab) have been established as a diagnostic biomarker (3–5). Because NMOSD is a lifelong disease characterized by unpredictable attacks, subsequent severe neurological disability, and variable responses to treatments, blood biomarkers for monitoring and predicting the course of the disease would be useful (6–8). Serum AQP4-Ab titers may serve as such a disease biomarker; however, they have failed to show consistent results regarding their correlations with disease activity, severity, outcome, or responses to therapy (9–14). Currently, no blood biomarkers for monitoring are available in clinical practice.

Glial fibrillary acidic protein (GFAP) is the specific intermediate filament protein that constitutes the cytoskeleton of astrocytes (15). Damaged astrocytes release GFAP into interstitial fluid, cerebrospinal fluid (CSF), and finally the blood. Because NMOSD is an astrocytopathy, GFAP blood levels may be a useful biomarker for NMOSD. The recent development of ultrasensitive single-molecule array (Simoa) technology has expedited the realization of the potential of GFAP as a biomarker for NMOSD (16, 17).

In this review article, we will first briefly provide basic information about GFAP protein and its function in the brain. Then, we will review detection methods for GFAP protein in the blood and the recent evidence for the potential of GFAP as a blood biomarker for NMOSD. Finally, we will discuss several considerations in using GFAP as a disease biomarker and future directions.

## GFAP

GFAP, a type III intermediate filament protein that was discovered by Dr. Eng in 1969 (18, 19), is responsible for the main cytoskeletal structure of astrocytes (19, 20). Apart from being present in the CNS, GFAP is also present in non-myelinated Schwann cells in the peripheral nervous system (PNS) and in enteric glia cells, which constitute the enteric nervous system (21, 22).

**Figure 1:**
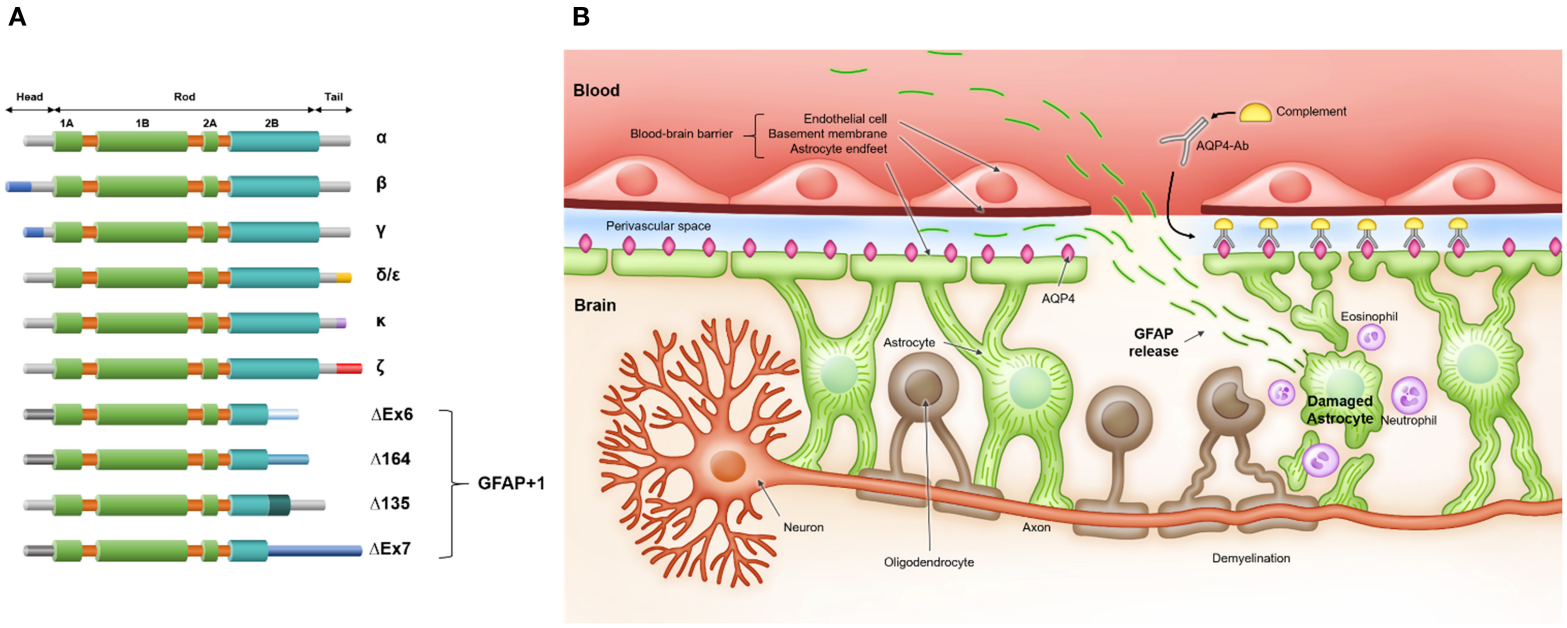
**(A)** Glial fibrillary acid protein (GFAP) isoforms and **(B)** release of GFAP after astrocyte injury in neuromyelitis optica spectrum disorder. **(A)** GFAP protein consists of three domains: N-terminal head, central rod, and C-terminal tail. The head domain is crucial for filament assembly, the rod domain has a role in filament formation by coiling between polypeptides, and the tail domain is important in stabilizing the intermediate filament. **(B)** Serum anti-aquaporin-4 antibodies (AQP4-Ab) penetrate the blood-brain barrier and bind to aquaporin-4 (AQP4) on astrocyte endfeet. Antibody- and complement-dependent cellular cytotoxicity results in inflammatory cell recruitment, astrocyte damage, demyelination, and neuronal loss. After astrocyte damage, GFAP, an astrocytic scaffold protein, is released into interstitial and cerebrospinal fluid and finally reaches the blood through an impaired blood-brain barrier and/or glymphatic efflux.

The human GFAP gene consists of nine exons and is located on chromosome 17 (17q21), spanning 10 kb (23). Alternative splicing occurs, and several GFAP isoforms have been identified (Figure 1A) (24). The three major domains of GFAP protein are the head, rod, and tail domains. The head domain is followed by the rod domain, which is composed of four α-helical coils. The N-terminal head domain is crucial for filament assembly, the rod domain plays a role in filament formation by coiling between polypeptides, and the C-terminal tail domain is important for stabilizing intermediate filaments (25). GFAP-α is the most abundant isoform in the brain and spinal cord but is also present in the PNS (26). This is the most commonly detected and analyzed isoform in the literature (20). GFAP-β is primarily expressed in non-myelinated Schwann cells in the PNS and has an alternate N-terminal (27). GFAP-γ also has an alternative N-terminal and is mainly located in the corpus callosum (28). GFAP-δ/ε is specifically expressed in neurogenic niches, such as the subventricular zone, and has an alternate C-terminal known to interact with presenilin (29–31). In addition, GFAP-δ/ε plays a role in modulating intermediate filament network dynamics (24, 32). GFAP-κ and GFAP-ζ also have distinct alternative C-terminals, which can modulate the properties of intermediate filaments (31, 33). Furthermore, an additional four isoforms of GFAP are collectively called GFAP+1, indicating isoform formation by a single nucleotide frameshift. GFAP+1 is found in a limited number of astrocytes in patients with Alzheimer's disease, Down syndrome, and chronic epilepsy; however, its implications remain to be elucidated (34–36). Although the precise functions of the different isoforms are not well-known, these isoforms seem to play a role in modulating intermediate filament networks during physiological and pathological states (37).

GFAP serves numerous pivotal functions in the CNS. GFAP is important for maintaining the mechanical strength of astrocytes and supporting neighboring neurons (38). In addition, GFAP participates in astrocytic motility and mitosis (39–41), maintains the integrity of the blood-brain barrier (BBB) and myelination (42, 43), protects neurons against neurotransmitter excess (44, 45) and injury (46, 47), regulates vesicle trafficking and autophagy (48, 49), and promotes synaptic plasticity (50, 51). Because GFAP is a major structural scaffold of astrocytes, damaged astrocytes release GFAP into their environment, e.g., interstitial fluid and CSF. Such released GFAP finally reaches the blood through an impaired BBB and/or glymphatic efflux (Figure 1B) (52–56). As such, blood GFAP exhibits much potential as a biomarker reflecting the state of NMOSD.

## Ultrasensitive Detection of GFAP: Single-Molecule Array

GFAP concentrations can be detected with immunoassays such as enzyme-linked immunosorbent assay (ELISA) (57, 58). Conventional ELISA typically measures proteins at concentrations above 10^−12^ M (16). However, its sensitivity may be insufficient to reliably measure GFAP in the blood, of which concentrations in most patients with neurological disorders range from 10^−14^ to 10^−10^ M (0.5–5,000 pg/mL) (59–64). In patients with demyelinating diseases, the median CSF GFAP level is 8,601 pg/mL and the median serum GFAP level is 167 pg/mL from NMOSD and MS patient (59). The limit of quantification of commercial ELISA varies from 62.5 pg/mL (Eagle Biosciences, NH, USA) to 1,500 pg/mL (MilliporeSigma, MA, USA). Accordingly, although conventional ELISA measured CSF GFAP levels that showed promise as a potential biomarker for NMOSD (65–67), the blood GFAP levels demonstrated inconsistent results, indicating little clinical value for NMOSD (68, 69).

Recently, an ultrasensitive digital ELISA technology, Simoa, has been developed (16). The technique detects fluorescent signals from each single protein molecule by using femtoliter-volume chambers that isolate a single bead holding an immuno-complex with an enzymatic reporter generating fluorescence. High sensitivity to enzyme labeling and low background signals due to digitizing the detection of proteins has enabled the technology to detect blood proteins at subfemtomolar concentrations (<10^−15^ M) (16). There are also other quantifying methods for GFAP such as electrochemiluminescence-based immunoassays and mass spectrometry (70). However, Simoa not only requires the smallest amount (only femtoliters) of blood for testing, but also shows the best analytical sensitivity with the limit of quantification of serum GFAP of 0.467 pg/mL (71). The reliability of Simoa for detecting blood neuronal and glial proteins is also high, as shown by the strong correlations between CSF and serum levels measured by Simoa technology (59, 72).

## GFAP in Blood as a Biomarker for NMOSD

Recently, several studies have demonstrated that blood GFAP levels measured by Simoa have potential as a useful NMOSD biomarker for (1) differentiating NMOSD from other demyelinating diseases, (2) identifying and predicting clinical attacks, (3) monitoring disease disability and progression, and (4) evaluating treatment effects (59, 71, 73–78) (Table 1).

**Table 1:** GFAP in blood as a biomarker for NMOSD.

**Author**	Comparison of levels^*^			Attack vs. remission^*^	**Correlation with**		**Prediction for future attack**	**Treatment effect**
	**vs. HC**	**vs. MS**	**vs. MOGAD**		**Age**	**EDSS**	**(Elevated vs. non-elevated)**	
Watanabe et al. (59)	↑ (207.7 vs. 97.2)	↑ (207.7 vs. 121.1)	N/A	↑ (540.9 vs. 152.9)	NS	+	N/A	N/A
Kim et al. (73)	N/A	N/A	↑ (123.1 vs. 90.2)	↑ (253.8 vs. 104.4)	NS	+	N/A	N/A
Aktas et al. (75)	↑ (128.3 vs. 71.3)	↑ (128.3 vs. 97.5)	N/A	↑ (2,160 vs. 168.4)	+	N/A	Hazard ratio 3.09	Inebilizumab^†^
Schindler et al. (76)	NS (109.2 vs. 67.7)	N/A	NS (109.2 vs. 81.1)	N/A	+	+	Hazard ratio 11.6	N/A
Kim et al. (71)	↑ (154.1 vs. 98.9)	N/A	N/A	↑ (275.5 vs. 153.7)	NS	N/A	N/A	Rituximab^¶^
Chang et al. (77)	↑ (274.1 vs. 61.4)	↑ (274.1 vs. 66.5)	NS (274.1 vs. 136.7)	↑ (284.4 vs. 147.1)	NS	+	N/A	N/A
Zhang et al. (78)	↑ (149.7 vs. 68.7)	N/A	N/A	↑ (2,691 vs. 114.0)	NS	+	N/A	Tocilizumab, rituximab^§^

*EDSS, expanded disability status scale; GFAP, glial fibrillary acidic protein; HC, healthy control; MOGAD, myelin oligodendrocyte glycoprotein antibody-associated disease; MS, multiple sclerosis; N/A, not available; NMOSD, neuromyelitis optica spectrum disorder; NS, not significant*.

* *The unit for GFAP levels is pg/mL. The figures in parentheses are median level of blood GFAP of each group*.

† *Inebilizumab attenuated the attack-related increase in serum GFAP levels [inebilizumab, median fold change (FC): 1.1 vs. placebo, median FC: 20.2], and decreased serum GFAP levels in patients who did not experience attacks (inebilizumab, −12.9% vs. placebo, +2.9% at week 16)*.

¶ *Rituximab-treated patients manifested stable serum GFAP levels over time, but other immunosuppressant-treated patients, treated with corticosteroids and/or immunosuppressants (azathioprine, mycophenolate mofetil, or methotrexate), showed significantly increased serum GFAP levels over time (rituximab: baseline 145.6 pg/mL → follow-up 168.1 pg/mL, p = 0.433; immunosuppressant: baseline 128.6 pg/mL → follow-up 153.0 pg/mL, p < 0.001)*.

§ *Tocilizumab and rituximab decreased plasma GFAP levels by 36 and 23%, respectively, compared to the change between baseline and follow up of the prednisone-treated group*.

### Differentiating NMOSD From Other Demyelinating Diseases

It is important in clinical practice to differentiate NMOSD from other demyelinating diseases, including multiple sclerosis (MS) and myelin oligodendrocyte glycoprotein antibody-associated disease (MOGAD), because treatments for these diseases differ considerably. Inappropriate treatments may result in poor outcomes. For example, treating NMOSD patients with therapies for MS could worsen the disease (79–81). Although the testing of AQP4-Ab is essential for the diagnosis of NMOSD, differentiation of the diseases remains crucial. Contrary to the high specificity of the AQP4-Ab assay (96.6–99.8%), the sensitivity of the AQP4-Ab assay (48.7–76.7%) varies according to the assay methodology, indicating a high risk of false-negative results (82). In addition, a patient's treatment and clinical status can affect the result of an antibody assay (83, 84).

Serum GFAP levels could be used as a diagnostic marker for NMOSD, as they are significantly higher in NMOSD patients compared to those in healthy controls (59, 71, 75, 77, 78) and patients with other demyelinating diseases (MS or MOGAD) (59, 73, 75, 77). These findings are in line with immunopathological studies which showed that GFAP-positive astrocytes are highly destroyed only in active lesions of NMOSD but not in those of MS (85–90). Neurofilament light chain (NfL), a scaffolding protein of the neuronal cytoskeleton that is released upon axonal damage, may represent another diagnostic biomarker because it is also elevated in the blood of NMOSD patients, compared to healthy controls (59, 71, 75, 77, 78). However, serum NfL levels do not differ between NMOSD patients and MS or MOGAD patients (59, 73, 77), suggesting that NfL lacks specificity as a biomarker for NMOSD. A recent study proposed that the serum GFAP/NfL quotient at attack state could be a useful biomarker that differentiates NMOSD from MS with a sensitivity of 73.0% and a specificity of 75.8% (59). The serum GFAP/NfL quotient also distinguished AQP4-Ab-seropositive NMOSD from MOGAD and MS (77).

### Identifying and Predicting Clinical Attacks

Identifying and predicting clinical attacks in NMOSD patients would be useful. Attack or relapse is defined as new or worsening neurological symptoms with an objective sign on neurological examination correlating with new or aggravating magnetic resonance image (MRI) lesions (91). However, pseudo-attacks or pseudo-relapses, i.e., clinical exacerbations with similar symptoms and signs but without true lesions, also occur in NMOSD patients, and clinically distinguishing between the two conditions can be difficult (91). Furthermore, although currently no method can predict future clinical attacks, a recent report revealed that clinically silent MRI lesions may represent a high risk of relapse (92). However, clinically silent brain or spinal cord lesions are rare in NMOSD patients, and thus performing regular MRI to predict future relapses would be inefficient.

Serum GFAP levels may help identify and predict clinical attacks in NMOSD patients, as they are higher in the attack state than in the remission state, and their elevation is associated with recent relapses (59, 71, 73–75, 77, 78). In a longitudinal NMOSD cohort (median follow up: 17 months), serum GFAP levels alone successfully discriminated clinical attacks from remission with a sensitivity of 94.7% and a specificity of 74.6% (area under the receiver characteristic curve = 0.876). Remarkably, this performance was better than that of other blood biomarkers, such as NfL and the GFAP/NfL quotient (71). In line with this, another study on a longitudinal NMOSD cohort (median follow up: 12 months) showed that plasma GFAP levels were the most powerful contributor in a random forest model to differentiate relapses from remissions, compared to other biomarkers (NfL, GFAP, and GFAP/NfL) and clinical variables [age, annual relapse rates, expanded disability status scale (EDSS) score, disease duration, and treatment status] (78). After relapse, serum GFAP levels decrease over time, and most patients show reduced serum GFAP levels below the predefined cut-off value (≥3 standard deviations of mean levels in age-/sex-matched healthy controls) within 3 months (71, 74).

Notably, increased serum GFAP levels may indicate forthcoming clinical relapses. In a substudy of the N-MOmentum study, significantly increased serum GFAP levels were already observed 1 week before a clinical attack (93), and serum GFAP levels were linearly correlated with the risk of an upcoming attack (75). Additionally, patients with elevated serum GFAP levels at baseline (≥2 standard deviations of the mean level of healthy controls) showed a 3-fold higher risk of having future NMOSD attacks than patients without elevated serum GFAP levels at baseline (75). Similar results were shown by another study on a prospective longitudinal cohort. NMOSD patients with high serum GFAP levels (>90 pg/mL, the cut-off value was derived from the 75th percentile of serum GFAP levels in healthy controls) at baseline had a shorter time to a future attack than those without [adjusted hazard ratio (95% confidence interval):11.6 (1.3–105.6)] (76). Conversely, in the same NMOSD cohort, baseline serum NfL levels were not significantly associated with a risk of future attack (76).

### Monitoring Disease Disability and Progression

Monitoring disease disability is necessary to determine the severity and track the progression of the disease, and to assess treatment effectiveness (94). The most popular and widely used instrument is the EDSS. However, considering that the inter-rater variability of EDSS is as high as 30%, establishing an objective and easily measurable biomarker would be preferable (95). Many studies have demonstrated that blood GFAP concentration is independently associated with EDSS score in NMOSD patients (59, 73, 76–78, 96). Serum GFAP levels are also correlated with other clinical disability parameters, including the MS functional composite score, 9-Hole Peg Test, and paced auditory serial addition test (76). Blood NfL levels also tend to increase with EDSS score in NMOSD patients. However, the degree of association is not as strong as that of blood GFAP levels; positive correlations were significant in some studies (59, 77, 78, 96) but not in others (73, 76).

Serum GFAP levels may also be useful to monitor disease progression. NMOSD is considered to lack subclinical disease activity, and all disabilities are related to attacks (97, 98). Conversely, MS exhibits subclinical progression (99, 100). However, recent optical coherent tomography and visual evoked potential studies suggested subclinical neurodegeneration in NMOSD patients (101, 102). More recently, silent progression of brain atrophy was documented in NMOSD patients, even in clinically inactive patients (103). Additional studies on blood GFAP levels further support the concept of ongoing subclinical neurodegeneration in NMOSD. First, median blood GFAP levels during remission periods are significantly higher in NMOSD patients than those in healthy controls (59, 71, 75, 77, 78). Second, blood GFAP levels gradually increase over time even in patients with no clinical relapse, and the rate of increase of GFAP levels is faster than that related to normal aging (71). Third, monoclonal antibody treatments such as inebilizumab, tocilizumab, and rituximab decrease serum GFAP levels more than treatments with placebo or prednisolone (75, 78). This indicates that gradual increases in GFAP levels may reflect ongoing pathological processes and may be alleviated by active treatment. However, it should be noted that most of these findings have been derived from small studies conducted at single centers or from substudies of clinical trials that may be different from real clinical situations. Future larger studies are warranted to confirm these findings.

### Evaluating Treatment Effects

It would be useful to have blood markers as objective endpoints in determining therapeutic effects, as shown in a recent clinical trial (104), or as index markers for selecting optimal personalized treatments (105). Recent data suggest that blood GFAP levels may represent such markers. In a longitudinal follow-up study, rituximab (anti-CD20 monoclonal antibody)-treated NMOSD patients exhibited stable serum GFAP levels over time, in contrast to patients with other immunosuppressant treatments who showed significantly increased serum GFAP levels during the same period (71). Inebilizumab, an anti-CD19 monoclonal antibody, also prevented increases in serum GFAP levels. It attenuated the attack-related increase in serum GFAP levels (75) and significantly decreased serum GFAP levels in patients who did not experience an attack, as compared to placebo treatment (75). Tocilizumab, an anti-IL6 monoclonal antibody, also significantly reduced plasma GFAP levels in NMOSD patients, as compared to prednisolone (78). These findings are remarkable because they indicate that blood GFAP levels can reflect treatment responses during silent periods without clinical relapses.

## Special Considerations

### Age

Physiological aging gradually affects the brain (106), and serum GFAP levels increase with aging in healthy controls (59, 71, 75, 77). However, this positive association has not been consistently demonstrated in NMOSD patients (59, 71, 73, 75–78). One explanation for such inconsistent GFAP–age correlations could be that NMOSD patients tend to have high serum GFAP levels even at a young age. Furthermore, aging-related processes, such as increased astrogliosis, also appear to affect the clinical implication of GFAP in elderly patients. In a study that analyzed the effect of age on serum biomarkers in NMOSD patients, positive GFAP–EDSS correlations were distinctively stronger in the youngest (≤45 years) compared to the oldest (≥55 years) group (96). The association between GFAP levels and disease severity may have been compromised in elderly patients due to increased astrogliosis following neurodegeneration (96). Therefore, age should be considered when interpreting blood levels of neuronal and glial proteins in NMOSD patients.

### Temporal Trajectories

The temporal dynamics of GFAP and date of blood sampling are also important. After brain injury, the serum GFAP levels peak at 20 h and decline over 72 h, indicating estimated half-life as 24–48 h (107, 108). It should also be noted that GFAP levels increases from 1 week before the advent of clinical symptoms (75). Even detected during the remission state, GFAP levels in NMOSD patients are still higher than healthy controls (59, 77, 78). In reflecting acute NMOSD attacks, GFAP may represent the event most appropriately within 7 days after attack, since 92% samples drawn within 1–7 days following attacks showed elevated level of blood GFAP (≥2 standard deviations of mean level of healthy controls) (75).

### Specificity

Blood GFAP levels increase not only in NMOSD but in various neurological diseases (109), thus the specificity of GFAP as an NMOSD biomarker should be discussed. Blood GFAP levels in patients with NMOSD, which often increase more than 1,000 pg/mL during relapses, tend to be higher than in patients with other diseases such as relapsing remitting MS (59, 60, 77), progressive MS (59–61), and even ischemic stroke (62). This is because patients with NMOSD are accompanied by direct damage of astrocytes. However, blood GFAP levels can also increase very high in glioblastoma, traumatic brain injury, and hemorrhagic stroke, as the level of NMOSD during relapses (63, 64, 110, 111). Therefore, it is difficult to regard that blood GFAP levels alone are a pathognomonic biomarker for NMOSD. Another parameter like GFAP/NfL ratio may enhance the specificity in terms of discriminating NMOSD from other diseases (59). However, it should also be emphasized that GFAP alone reflects well the longitudinal disease course of NMOSD and may be the most appropriate marker to monitor the disease changes within the NMOSD cohort (71).

### NfL

As a representative biomarker of neuronal damage, serum NfL has also demonstrated disease association with NMOSD as well as MS (59, 71, 73, 76–78, 112, 113). However, serum NfL was not useful to distinguish NMOSD from other demyelinating diseases, and less sensitive and specific than serum GFAP in identifying and predicting NMOSD relapses (59, 71, 76–78). The value of NfL may be more pronounced elsewhere. Given that NfL is a neuronal structural component, serum NfL might be a better biomarker for monitoring the degree of neurodegeneration of NMOSD (101–103) and associated cognitive impairment (113) than serum GFAP. This possibility should be elucidated in future studies.

## Outlook

For GFAP to be used as a biomarker in clinical practice, several limitations that hinder the applicability of blood GFAP in clinical settings should be addressed. First, standard protocols and quality control criteria should be established across different laboratories (113). In addition, age-specific and sex-specific reference should also be developed. The dynamics of GFAP after releases upon NMOSD attacks should be explored to determine accurate blood GFAP half-life. This work should be paralleled with unraveling mechanisms and pathways of GFAP released from brain into the blood. Finally, the intervals for testing blood GFAP levels and guidelines for biomarker-based decision making should also be established.

Clinically, management strategies could be available by stratifying the risk of future attack based on both age-adjusted cut-off values and intraindividual changes in blood GFAP levels. Based on an individual's different strata of attack risk, clinicians could decide treatment initiation, continuation, and escalation/de-escalation of NMOSD patients. For example, it could be possible to set a serum GFAP range for the treatment response of patients and classify patients into treatment-responsive and treatment-resistant groups. This classification would enable precision treatment strategies that quickly change from one option to another suitable before it is too late (e.g., the advent of clinical relapses).

## Conclusions

Although more than 50 years have passed since GFAP was first discovered, only recently has GFAP been suggested as a reliable blood biomarker in clinical practice. The role of GFAP as a biomarker for NMOSD shows promise because GFAP not only has pathophysiological specificity that can reflect astrocytopathy as much as AQP4-Ab, but it also has the advantage of being quantifiable with much more sensitivity than AQP4-Ab. After several clinical and technical issues are resolved, blood GFAP levels may expedite the process of personalized care of NMOSD patients.

## Author Contributions

HK and E-JL contributed to conception and design of the review. HK wrote the manuscript. E-JL acquired funding. Y-ML, K-KK, and E-JL supervised the study and revised the manuscript. All authors contributed to manuscript revision, read, and approved the submitted version.

## Funding

This study was supported by grants from the Ministry of Science and ICT, South Korea (NRF−2018R1C1B6008884).

## Conflict of Interest

The authors declare that the research was conducted in the absence of any commercial or financial relationships that could be construed as a potential conflict of interest.

## Publisher's Note

All claims expressed in this article are solely those of the authors and do not necessarily represent those of their affiliated organizations, or those of the publisher, the editors and the reviewers. Any product that may be evaluated in this article, or claim that may be made by its manufacturer, is not guaranteed or endorsed by the publisher.

## References

[1] Papadopoulos MC Verkman AS. Aquaporin 4 and neuromyelitis optica. Lancet Neurol. (2012) 11:535–44. 10.1016/S1474-4422(12)70133-3 22608667 PMC3678971

[2] Fujihara K. Neuromyelitis optica spectrum disorders: still evolving and broadening. Curr Opin Neurol. (2019) 32:385–94. 10.1097/WCO.0000000000000694 30893099 PMC6522202

[3] Lennon VA Kryzer TJ Pittock SJ Verkman AS Hinson SR. IgG marker of optic-spinal multiple sclerosis binds to the aquaporin-4 water channel. J Exp Med. (2005) 202:473–7. 10.1084/jem.20050304 16087714 PMC2212860

[4] Lennon VA Wingerchuk DM Kryzer TJ Pittock SJ Lucchinetti CF Fujihara K. A serum autoantibody marker of neuromyelitis optica: distinction from multiple sclerosis. Lancet. (2004) 364:2106–12. 10.1016/S0140-6736(04)17551-X 15589308

[5] Papadopoulos MC Bennett JL Verkman AS. Treatment of neuromyelitis optica: state-of-the-art and emerging therapies. Nat Rev Neurol. (2014) 10:493–506. 10.1038/nrneurol.2014.141 25112508 PMC4229040

[6] Melamed E Levy M Waters PJ Sato DK Bennett JL John GR. Update on biomarkers in neuromyelitis optica. Neurol Neuroimmunol Neuroinflamm. (2015) 2:e134. 10.1212/NXI.0000000000000134 26236760 PMC4516398

[7] Lee EJ Lim YM Kim SY Lee J Kim H Jin JY. The clinical and prognostic value of antinuclear antibodies in NMO-IgG seropositive neuromyelitis optica spectrum disorder. J Neuroimmunol. (2019) 328:1–4. 10.1016/j.jneuroim.2018.11.012 30543869

[8] Kim S Lee EJ Kim KW Seo D Moon S Kim KK. Quality of life of patients with multiple sclerosis and neuromyelitis optica spectrum disorders: cross-sectional and longitudinal analysis. Mult Scler Relat Disord. (2022) 58:103500. 10.1016/j.msard.2022.103500 35032884

[9] Jitprapaikulsan J Fryer JP Majed M Smith CY Jenkins SM Cabre P. Clinical utility of AQP4-IgG titers and measures of complement-mediated cell killing in NMOSD. Neurol Neuroimmunol Neuroinflam. (2020) 7:e727. 10.1212/NXI.0000000000000727 PMC7286655 35413004

[10] Takahashi T Fujihara K Nakashima I Misu T Miyazawa I Nakamura M. Anti-aquaporin-4 antibody is involved in the pathogenesis of NMO: a study on antibody titre. Brain. (2007) 130:1235–43. 10.1093/brain/awm062 17449477

[11] Akaishi T Takahashi T Nakashima I Abe M Ishii T Aoki M. Repeated follow-up of AQP4-IgG titer by cell-based assay in neuromyelitis optica spectrum disorders (NMOSD). J Neurol Sci. (2020) 410:116671. 10.1016/j.jns.2020.116671 31927341

[12] Jarius S Aboul-Enein F Waters P Kuenz B Hauser A Berger T. Antibody to aquaporin-4 in the long-term course of neuromyelitis optica. Brain. (2008) 131:3072–80. 10.1093/brain/awn240 18945724 PMC2577801

[13] Dujmovic I Mader S Schanda K Deisenhammer F Stojsavljevic N Kostic J. Temporal dynamics of cerebrospinal fluid anti-aquaporin-4 antibodies in patients with neuromyelitis optica spectrum disorders. J Neuroimmunol. (2011) 234:124–30. 10.1016/j.jneuroim.2011.01.007 21316112

[14] Takahashi T Miyazawa I Misu T Takano R Nakashima I Fujihara K. Intractable hiccup and nausea in neuromyelitis optica with anti-aquaporin-4 antibody: a herald of acute exacerbations. J Neurol Neurosurg Psychiatry. (2008) 79:1075–8. 10.1136/jnnp.2008.145391 18420727

[15] Brenner M Johnson AB Boespflug-Tanguy O Rodriguez D Goldman JE Messing A. Mutations in GFAP, encoding glial fibrillary acidic protein, are associated with Alexander disease. Nat Genet. (2001) 27:117–20. 10.1038/83679 11138011

[16] Rissin DM Kan CW Campbell TG Howes SC Fournier DR Song L. Single-molecule enzyme-linked immunosorbent assay detects serum proteins at subfemtomolar concentrations. Nat Biotechnol. (2010) 28:595–9. 10.1038/nbt.1641 20495550 PMC2919230

[17] Chang L Rissin DM Fournier DR Piech T Patel PP Wilson DH. Single molecule enzyme-linked immunosorbent assays: theoretical considerations. J Immunol Methods. (2012) 378:102–15. 10.1016/j.jim.2012.02.011 22370429 PMC3327511

[18] Eng L Gerstl B Vanderhaeghen J. A study of proteins in old multiple sclerosis plaques. Trans Am Soc Neurochem. (1970) 1:42.2512757

[19] Eng LF Ghirnikar RS Lee YL. Glial fibrillary acidic protein: GFAP-thirty-one years (1969-2000). Neurochem Res. (2000) 25:1439–51. 10.1023/A:1007677003387 11059815

[20] Hol EM Capetanaki Y. Type III intermediate filaments desmin, glial fibrillary acidic protein (GFAP), vimentin, and peripherin. Cold Spring Harb Perspect Biol. (2017) 9:a021642. 10.1101/cshperspect.a021642 29196434 PMC5710105

[21] Gulbransen BD Sharkey KA. Novel functional roles for enteric glia in the gastrointestinal tract. Nat Rev Gastroenterol Hepatol. (2012) 9:625–32. 10.1038/nrgastro.2012.138 22890111

[22] Laranjeira C Sandgren K Kessaris N Richardson W Potocnik A Vanden Berghe P. Glial cells in the mouse enteric nervous system can undergo neurogenesis in response to injury. J Clin Invest. (2011) 121:3412–24. 10.1172/JCI58200 21865647 PMC3163972

[23] Blechingberg J Lykke-Andersen S Jensen TH Jørgensen AL Nielsen AL. Regulatory mechanisms for 3'-end alternative splicing and polyadenylation of the Glial Fibrillary Acidic Protein, GFAP, transcript. Nucleic Acids Res. (2007) 35:7636–50. 10.1093/nar/gkm931 17981838 PMC2190720

[24] Moeton M Stassen OM Sluijs JA van der Meer VW Kluivers LJ van Hoorn H. GFAP isoforms control intermediate filament network dynamics, cell morphology, and focal adhesions. Cell Mol Life Sci. (2016) 73:4101–20. 10.1007/s00018-016-2239-5 27141937 PMC5043008

[25] Ralton JE Lu X Hutcheson AM Quinlan RA. Identification of two N-terminal non-alpha-helical domain motifs important in the assembly of glial fibrillary acidic protein. J Cell Sci. (1994) 107:1935–48. 10.1242/jcs.107.7.1935 7983160

[26] Reeves SA Helman LJ Allison A Israel MA. Molecular cloning and primary structure of human glial fibrillary acidic protein. Proc Natl Acad Sci USA. (1989) 86:5178–82. 10.1073/pnas.86.13.5178 2740350 PMC297581

[27] Galea E Dupouey P Feinstein DL. Glial fibrillary acidic protein mRNA isotypes: expression *in vitro* and *in vivo*. J Neurosci Res. (1995) 41:452–61. 10.1002/jnr.490410404 7473876

[28] Zelenika D Grima B Brenner M Pessac B. A novel glial fibrillary acidic protein mRNA lacking exon 1. Brain Res Mol Brain Res. (1995) 30:251–8. 10.1016/0169-328X(95)00010-P 7637576

[29] Roelofs RF Fischer DF Houtman SH Sluijs JA Van Haren W Van Leeuwen FW. Adult human subventricular, subgranular, and subpial zones contain astrocytes with a specialized intermediate filament cytoskeleton. Glia. (2005) 52:289–300. 10.1002/glia.20243 16001427

[30] Nielsen AL Holm IE Johansen M Bonven B Jørgensen P Jørgensen AL. A new splice variant of glial fibrillary acidic protein, GFAP epsilon, interacts with the presenilin proteins. J Biol Chem. (2002) 277:29983–91. 10.1074/jbc.M112121200 12058025

[31] Kamphuis W Mamber C Moeton M Kooijman L Sluijs JA Jansen AH. GFAP isoforms in adult mouse brain with a focus on neurogenic astrocytes and reactive astrogliosis in mouse models of Alzheimer disease. PLoS ONE. (2012) 7:e42823. 10.1371/journal.pone.0042823 22912745 PMC3418292

[32] Perng MD Wen SF Gibbon T Middeldorp J Sluijs J Hol EM. Glial fibrillary acidic protein filaments can tolerate the incorporation of assembly-compromised GFAP-delta, but with consequences for filament organization and alphaB-crystallin association. Mol Biol Cell. (2008) 19:4521–33. 10.1091/mbc.e08-03-0284 18685083 PMC2555932

[33] Blechingberg J Holm IE Nielsen KB Jensen TH Jørgensen AL Nielsen AL. Identification and characterization of GFAPkappa, a novel glial fibrillary acidic protein isoform. Glia. (2007) 55:497–507. 10.1002/glia.20475 17203480

[34] Kamphuis W Middeldorp J Kooijman L Sluijs JA Kooi EJ Moeton M. Glial fibrillary acidic protein isoform expression in plaque related astrogliosis in Alzheimer's disease. Neurobiol Aging. (2014) 35:492–510. 10.1016/j.neurobiolaging.2013.09.035 24269023

[35] Hol EM Roelofs RF Moraal E Sonnemans MA Sluijs JA Proper EA. Neuronal expression of GFAP in patients with Alzheimer pathology and identification of novel GFAP splice forms. Mol Psychiatry. (2003) 8:786–96. 10.1038/sj.mp.4001379 12931206

[36] Boer K Middeldorp J Spliet WG Razavi F van Rijen PC Baayen JC. Immunohistochemical characterization of the out-of frame splice variants GFAP Delta164/Deltaexon 6 in focal lesions associated with chronic epilepsy. Epilepsy Res. (2010) 90:99–109. 10.1016/j.eplepsyres.2010.03.014 20430588

[37] Middeldorp J Hol EM. GFAP in health and disease. Prog Neurobiol. (2011) 93:421–43. 10.1016/j.pneurobio.2011.01.005 21219963

[38] Yang Z Wang KK. Glial fibrillary acidic protein: from intermediate filament assembly and gliosis to neurobiomarker. Trends Neurosci. (2015) 38:364–74. 10.1016/j.tins.2015.04.003 25975510 PMC4559283

[39] Elobeid A Bongcam-Rudloff E Westermark B Nister M. Effects of inducible glial fibrillary acidic protein on glioma cell motility and proliferation. J Neurosci Res. (2000) 60:245–56. 10.1002/(SICI)1097-4547(20000415)60:2<245::AID-JNR14>3.0.CO;2-1 10740230

[40] Triolo D Dina G Lorenzetti I Malaguti M Morana P Del Carro U. Loss of glial fibrillary acidic protein (GFAP) impairs Schwann cell proliferation and delays nerve regeneration after damage. J Cell Sci. (2006) 119:3981–93. 10.1242/jcs.03168 16988027

[41] Rutka JT Smith SL. Transfection of human astrocytoma cells with glial fibrillary acidic protein complementary DNA: analysis of expression, proliferation, and tumorigenicity. Cancer Res. (1993) 53:3624–31. 8339269

[42] Pekny M Stanness KA Eliasson C Betsholtz C Janigro D. Impaired induction of blood-brain barrier properties in aortic endothelial cells by astrocytes from GFAB-deficient mice. Glia. (1998) 22:390–400. 10.1002/(SICI)1098-1136(199804)22:4<390::AID-GLIA8>3.0.CO;2-7 9517571

[43] Liedtke W Edelmann W Bieri PL Chiu F-C Cowan NJ Kucherlapati R. GFAP is necessary for the integrity of CNS white matter architecture and long-term maintenance of myelination. Neuron. (1996) 17:607–15. 10.1016/S0896-6273(00)80194-4 8893019

[44] Lieth E Barber AJ Xu B Dice C Ratz MJ Tanase D. Glial reactivity and impaired glutamate metabolism in short-term experimental diabetic retinopathy. Penn State Retina Research Group. Diabetes. (1998) 47:815–20. 10.2337/diabetes.47.5.815 9588455

[45] Pekny M Eliasson C Siushansian R Ding M Dixon SJ Pekna M. The impact of genetic removal of GFAP and/or vimentin on glutamine levels and transport of glucose and ascorbate in astrocytes. Neurochem Res. (1999) 24:1357–62. 10.1023/A:1022572304626 10555775

[46] Nawashiro H Messing A Azzam N Brenner M. Mice lacking GFAP are hypersensitive to traumatic cerebrospinal injury. Neuroreport. (1998) 9:1691–6. 10.1097/00001756-199806010-00004 9665584

[47] Otani N Nawashiro H Fukui S Ooigawa H Ohsumi A Toyooka T. Enhanced hippocampal neurodegeneration after traumatic or kainate excitotoxicity in GFAP-null mice. J Clin Neurosci. (2006) 13:934–8. 10.1016/j.jocn.2005.10.018 17085299

[48] Bandyopadhyay U Sridhar S Kaushik S Kiffin R Cuervo AM. Identification of regulators of chaperone-mediated autophagy. Molecular Cell. (2010) 39:535–47. 10.1016/j.molcel.2010.08.004 20797626 PMC2945256

[49] Potokar M Kreft M Li L Daniel Andersson J Pangršič T Chowdhury HH. Cytoskeleton and vesicle mobility in astrocytes. Traffic. (2007) 8:12–20. 10.1111/j.1600-0854.2006.00509.x 17229312

[50] McCall M Gregg R Behringer R Brenner M Delaney C Galbreath E. Targeted deletion in astrocyte intermediate filament (Gfap) alters neuronal physiology. Proc Natl Acad Sci. (1996) 93:6361–6. 10.1073/pnas.93.13.6361 8692820 PMC39027

[51] Shibuki K Gomi H Chen L Bao S Kim JJ Wakatsuki H. Deficient cerebellar long-term depression, impaired eyeblink conditioning, and normal motor coordination in GFAP mutant mice. Neuron. (1996) 16:587–99. 10.1016/S0896-6273(00)80078-1 8785056

[52] Chodobski A Zink BJ Szmydynger-Chodobska J. Blood-brain barrier pathophysiology in traumatic brain injury. Transl Stroke Res. (2011) 2:492–516. 10.1007/s12975-011-0125-x 22299022 PMC3268209

[53] Obermeier B Daneman R Ransohoff RM. Development, maintenance and disruption of the blood-brain barrier. Nature Medicine. (2013) 19:1584–96. 10.1038/nm.3407 24309662 PMC4080800

[54] Da Mesquita S Fu Z Kipnis J. The meningeal lymphatic system: a new player in neurophysiology. Neuron. (2018) 100:375–88. 10.1016/j.neuron.2018.09.022 30359603 PMC6268162

[55] Mestre H Mori Y Nedergaard M. The brain's glymphatic system: current controversies. Trends Neurosci. (2020) 43:458–66. 10.1016/j.tins.2020.04.003 32423764 PMC7331945

[56] Plog BA Dashnaw ML Hitomi E Peng W Liao Y Lou N. Biomarkers of traumatic injury are transported from brain to blood via the glymphatic system. J Neurosci. (2015) 35:518–26. 10.1523/JNEUROSCI.3742-14.2015 25589747 PMC4293408

[57] Petzold A Keir G Green AJ Giovannoni G Thompson EJ. An ELISA for glial fibrillary acidic protein. J Immunol Methods. (2004) 287:169–77. 10.1016/j.jim.2004.01.015 15099765

[58] Rosengren LE Wikkelsø C Hagberg L. A sensitive ELISA for glial fibrillary acidic protein: application in CSF of adults. J Neurosci Methods. (1994) 51:197–204. 10.1016/0165-0270(94)90011-6 8051950

[59] Watanabe M Nakamura Y Michalak Z Isobe N Barro C Leppert D. Serum GFAP and neurofilament light as biomarkers of disease activity and disability in NMOSD. Neurology. (2019) 93:e1299–e311. 10.1212/WNL.0000000000008160 31471502

[60] Abdelhak A Huss A Kassubek J Tumani H Otto M. Serum GFAP as a biomarker for disease severity in multiple sclerosis. Sci Rep. (2018) 8:14798. 10.1038/s41598-018-33158-8 30287870 PMC6172254

[61] Ayrignac X Le Bars E Duflos C Hirtz C Maleska Maceski A Carra-Dallière C. Serum GFAP in multiple sclerosis: correlation with disease type and MRI markers of disease severity. Sci Rep. (2020) 10:10923. 10.1038/s41598-020-67934-2 32616916 PMC7331703

[62] Kalra LP Khatter H Ramanathan S Sapehia S Devi K Kaliyaperumal A. Serum GFAP for stroke diagnosis in regions with limited access to brain imaging (BE FAST India). Eur Stroke J. (2021) 6:176–84. 10.1177/23969873211010069 34414293 PMC8370074

[63] Jung CS Foerch C Schänzer A Heck A Plate KH Seifert V. Serum GFAP is a diagnostic marker for glioblastoma multiforme. Brain. (2007) 130:3336–41. 10.1093/brain/awm263 17998256

[64] Czeiter E Amrein K Gravesteijn BY Lecky F Menon DK Mondello S. Blood biomarkers on admission in acute traumatic brain injury: relations to severity, CT findings and care path in the CENTER-TBI study. EBioMedicine. (2020) 56:102785. 10.1016/j.ebiom.2020.102785 32464528 PMC7251365

[65] Uzawa A Mori M Sawai S Masuda S Muto M Uchida T. Cerebrospinal fluid interleukin-6 and glial fibrillary acidic protein levels are increased during initial neuromyelitis optica attacks. Clin Chim Acta. (2013) 421:181–3. 10.1016/j.cca.2013.03.020 23535508

[66] Misu T Takano R Fujihara K Takahashi T Sato S Itoyama Y. Marked increase in cerebrospinal fluid glial fibrillar acidic protein in neuromyelitis optica: an astrocytic damage marker. J Neurol Neurosurg Psychiatry. (2009) 80:575–7. 10.1136/jnnp.2008.150698 19372295

[67] Takano R Misu T Takahashi T Sato S Fujihara K Itoyama Y. Astrocytic damage is far more severe than demyelination in NMO: a clinical CSF biomarker study. Neurology. (2010) 75:208–16. 10.1212/WNL.0b013e3181e2414b 20644148

[68] Storoni M Verbeek MM Illes Z Marignier R Teunissen CE Grabowska M. Serum GFAP levels in optic neuropathies. J Neurol Sci. (2012) 317:117–22. 10.1016/j.jns.2012.02.012 22410258

[69] Storoni M Petzold A Plant GT. The use of serum glial fibrillary acidic protein measurements in the diagnosis of neuromyelitis optica spectrum optic neuritis. PLoS ONE. (2011) 6:e23489. 10.1371/journal.pone.0023489 21876753 PMC3158082

[70] Petzold A. Glial fibrillary acidic protein is a body fluid biomarker for glial pathology in human disease. Brain Res. (2015) 1600:17–31. 10.1016/j.brainres.2014.12.027 25543069

[71] Kim H Lee EJ Kim S Choi LK Kim HJ Kim HW. Longitudinal follow-up of serum biomarkers in patients with neuromyelitis optica spectrum disorder. Mult Scler. (2021) 0:13524585211024978. 10.1177/13524585211024978 34212756

[72] Högel H Rissanen E Barro C Matilainen M Nylund M Kuhle J. Serum glial fibrillary acidic protein correlates with multiple sclerosis disease severity. Mult Scler. (2020) 26:210–9. 10.1177/1352458518819380 30570436

[73] Kim H Lee EJ Kim S Choi LK Kim K Kim HW. Serum biomarkers in myelin oligodendrocyte glycoprotein antibody-associated disease. Neurol Neuroimmunol Neuroinflamm. (2020) 7:e708. 10.1212/NXI.0000000000000708 32184342 PMC7136043

[74] Hyun J-W Kim Y Kim SY Lee MY Kim S-H Kim HJ. Investigating the presence of interattack astrocyte damage in neuromyelitis optica spectrum disorder. Longitudinal analysis of serum glial fibrillary acidic protein. Neurol Neuroimmunol Neuroinflamm. (2021) 8:e965. 10.1212/NXI.0000000000000965 33846219 PMC8054958

[75] Aktas O Smith MA Rees WA Bennett JL She D Katz E. Serum glial fibrillary acidic protein: a neuromyelitis optica spectrum disorder biomarker. Ann Neurol. (2021) 89:895–910. 10.1002/ana.26067 33724534 PMC8252046

[76] Schindler P Grittner U Oechtering J Leppert D Siebert N Duchow AS. Serum GFAP and NfL as disease severity and prognostic biomarkers in patients with aquaporin-4 antibody-positive neuromyelitis optica spectrum disorder. J Neuroinflamm. (2021) 18:105. 10.1186/s12974-021-02138-7 33933106 PMC8088712

[77] Chang X Huang W Wang L ZhangBao J Zhou L Lu C. Serum neurofilament light and GFAP are associated with disease severity in inflammatory disorders with aquaporin-4 or myelin oligodendrocyte glycoprotein antibodies. Front Immunol. (2021) 12:647618. 10.3389/fimmu.2021.647618 33796113 PMC8008082

[78] Zhang T-X Chen J-S Du C Zeng P Zhang H Wang X. Longitudinal treatment responsiveness on plasma neurofilament light chain and glial fibrillary acidic protein levels in neuromyelitis optica spectrum disorder. Ther Adv Neurol Disord. (2021) 14:17562864211054952. 10.1177/17562864211054952 34777577 PMC8573482

[79] Kleiter I Hellwig K Berthele A Kümpfel T Linker RA Hartung H-P. Failure of natalizumab to prevent relapses in neuromyelitis optica. Arch Neurol. (2012) 69:239–45. 10.1001/archneurol.2011.216 22332191

[80] Min J-H Kim BJ Lee KH. Development of extensive brain lesions following fingolimod (FTY720) treatment in a patient with neuromyelitis optica spectrum disorder. Multiple Sclerosis J. (2012) 18:113–5. 10.1177/1352458511431973 22146605

[81] Palace J Leite MI Nairne A Vincent A. Interferon beta treatment in neuromyelitis optica: increase in relapses and aquaporin 4 antibody titers. Arch Neurol. (2010) 67:1016–7. 10.1001/archneurol.2010.188 20697055

[82] Waters PJ Pittock SJ Bennett JL Jarius S Weinshenker BG Wingerchuk DM. Evaluation of aquaporin-4 antibody assays. Clin Exp Neuroimmunol. (2014) 5:290–303. 10.1111/cen3.12107 27840658 PMC5102503

[83] Waters PJ McKeon A Leite MI Rajasekharan S Lennon VA Villalobos A. Serologic diagnosis of NMO: a multicenter comparison of aquaporin-4-IgG assays. Neurology. (2012) 78:665–71. 10.1212/WNL.0b013e318248dec1 22302543 PMC3286228

[84] Cohen M De Sèze J Marignier R Lebrun C. False positivity of anti aquaporin-4 antibodies in natalizumab-treated patients. Mult Scler. (2016) 22:1231–4. 10.1177/1352458516630823 26869528

[85] Takai Y Misu T Suzuki H Takahashi T Okada H Tanaka S. Staging of astrocytopathy and complement activation in neuromyelitis optica spectrum disorders. Brain. (2021) 144:2401–15. 10.1093/brain/awab102 33711152

[86] Misu T Höftberger R Fujihara K Wimmer I Takai Y Nishiyama S. Presence of six different lesion types suggests diverse mechanisms of tissue injury in neuromyelitis optica. Acta Neuropathol. (2013) 125:815–27. 10.1007/s00401-013-1116-7 23579868 PMC3661909

[87] Masaki K Suzuki SO Matsushita T Matsuoka T Imamura S Yamasaki R. Connexin 43 astrocytopathy linked to rapidly progressive multiple sclerosis and neuromyelitis optica. PLoS ONE. (2013) 8:e72919. 10.1371/journal.pone.0072919 23991165 PMC3749992

[88] Lucchinetti CF Mandler RN McGavern D Bruck W Gleich G Ransohoff RM. A role for humoral mechanisms in the pathogenesis of Devic's neuromyelitis optica. Brain. (2002) 125:1450–61. 10.1093/brain/awf151 12076996 PMC5444467

[89] Hayashida S Masaki K Yonekawa T Suzuki SO Hiwatashi A Matsushita T. Early and extensive spinal white matter involvement in neuromyelitis optica. Brain Pathol. (2017) 27:249–65. 10.1111/bpa.12386 27082714 PMC8029352

[90] Brück W Popescu B Lucchinetti CF Markovic-Plese S Gold R Thal DR. Neuromyelitis optica lesions may inform multiple sclerosis heterogeneity debate. Ann Neurol. (2012) 72:385–94. 10.1002/ana.23621 23034911

[91] Kessler RA Mealy MA Levy M. Early indicators of relapses vs pseudorelapses in neuromyelitis optica spectrum disorder. Neurol Neuroimmunol Neuroinflamm. (2016) 3:e269. 10.1212/NXI.0000000000000269 27508210 PMC4966291

[92] Camera V Holm-Mercer L Ali AAH Messina S Horvat T Kuker W. Frequency of new silent MRI lesions in myelin oligodendrocyte glycoprotein antibody disease and aquaporin-4 antibody neuromyelitis optica spectrum disorder. JAMA Network Open. (2021) 4:e2137833–e. 10.1001/jamanetworkopen.2021.37833 34878547 PMC8655599

[93] Cree BAC Bennett JL Kim HJ Weinshenker BG Pittock SJ Wingerchuk DM. Inebilizumab for the treatment of neuromyelitis optica spectrum disorder (N-MOmentum): a double-blind, randomised placebo-controlled phase 2/3 trial. Lancet. (2019) 394:1352–63. 10.1016/S0140-6736(19)31817-3 31495497

[94] Meyer-Moock S Feng Y-S Maeurer M Dippel F-W Kohlmann T. Systematic literature review and validity evaluation of the expanded disability status scale (EDSS) and the multiple sclerosis functional composite (MSFC) in patients with multiple sclerosis. BMC Neurol. (2014) 14:58. 10.1186/1471-2377-14-58 24666846 PMC3986942

[95] Cohen M Bresch S Thommel Rocchi O Morain E Benoit J Levraut M. Should we still only rely on EDSS to evaluate disability in multiple sclerosis patients? A study of inter and intra rater reliability. Multiple Sclerosis Relat Disord. (2021) 54:103144. 10.1016/j.msard.2021.103144 34274736

[96] Lee EJ Lim YM Kim S Choi L Kim H Kim K. Clinical implication of serum biomarkers and patient age in inflammatory demyelinating diseases. Ann Clin Transl Neurol. (2020) 7:992–1001. 10.1002/acn3.51070 32495489 PMC7317646

[97] Akaishi T Takahashi T Misu T Abe M Ishii T Fujimori J. Progressive patterns of neurological disability in multiple sclerosis and neuromyelitis optica spectrum disorders. Sci Rep. (2020) 10:13890. 10.1038/s41598-020-70919-w 32807848 PMC7431838

[98] Wingerchuk DM Pittock SJ Lucchinetti CF Lennon VA Weinshenker BG. A secondary progressive clinical course is uncommon in neuromyelitis optica. Neurology. (2007) 68:603–5. 10.1212/01.wnl.0000254502.87233.9a 17310032

[99] Correale J Gaitán MI Ysrraelit MC Fiol MP. Progressive multiple sclerosis: from pathogenic mechanisms to treatment. Brain. (2017) 140:527–46. 10.1093/brain/aww258 27794524

[100] Cree BAC Hollenbach JA Bove R Kirkish G Sacco S Caverzasi E. Silent progression in disease activity-free relapsing multiple sclerosis. Ann Neurol. (2019) 85:653–66. 10.1002/ana.25463 30851128 PMC6518998

[101] Ringelstein M Harmel J Zimmermann H Brandt AU Paul F Haarmann A. Longitudinal optic neuritis-unrelated visual evoked potential changes in NMO spectrum disorders. Neurology. (2020) 94:e407–18. 10.1212/WNL.0000000000008684 31796527

[102] Pisa M Ratti F Vabanesi M Radaelli M Guerrieri S Moiola L. Subclinical neurodegeneration in multiple sclerosis and neuromyelitis optica spectrum disorder revealed by optical coherence tomography. Mult Scler. (2020) 26:1197–206. 10.1177/1352458519861603 31392924

[103] Masuda H Mori M Hirano S Uzawa A Uchida T Muto M. Silent progression of brain atrophy in aquaporin-4 antibody-positive neuromyelitis optica spectrum disorder. J Neurol Neurosurg Psychiatry. (2022) 93:32–40. 10.1136/jnnp-2021-326386 34362853 PMC8685614

[104] Hauser SL Bar-Or A Cohen JA Comi G Correale J Coyle PK. Ofatumumab versus teriflunomide in multiple sclerosis. New Engl J Med. (2020) 383:546–57. 10.1056/NEJMoa1917246 32757523

[105] Landeck L Kneip C Reischl J Asadullah K. Biomarkers and personalized medicine: current status and further perspectives with special focus on dermatology. Exp Dermatol. (2016) 25:333–9. 10.1111/exd.12948 27167702

[106] Khalil M Pirpamer L Hofer E Voortman MM Barro C Leppert D. Serum neurofilament light levels in normal aging and their association with morphologic brain changes. Nat Commun. (2020) 11:812. 10.1038/s41467-020-14612-6 32041951 PMC7010701

[107] Papa L Brophy GM Welch RD Lewis LM Braga CF Tan CN. Time course and diagnostic accuracy of glial and neuronal blood biomarkers GFAP and UCH-L1 in a large cohort of trauma patients with and without mild traumatic brain injury. JAMA Neurol. (2016) 73:551–60. 10.1001/jamaneurol.2016.0039 27018834 PMC8805143

[108] Thelin EP Zeiler FA Ercole A Mondello S Büki A Bellander BM. Serial sampling of serum protein biomarkers for monitoring human traumatic brain injury dynamics: a systematic review. Front Neurol. (2017) 8:300. 10.3389/fneur.2017.00300 28717351 PMC5494601

[109] Abdelhak A Foschi M Abu-Rumeileh S Yue JK D'Anna L Huss A. Blood GFAP as an emerging biomarker in brain and spinal cord disorders. Nat Rev Neurol. (2022) 18:158–72. 10.1038/s41582-021-00616-3 35115728

[110] Huebschmann NA Luoto TM Karr JE Berghem K Blennow K Zetterberg H. Comparing glial fibrillary acidic protein (GFAP) in serum and plasma following mild traumatic brain injury in older adults. Front Neurol. (2020) 11:1054. 10.3389/fneur.2020.01054 33071938 PMC7530818

[111] Shahim P Gren M Liman V Andreasson U Norgren N Tegner Y. Serum neurofilament light protein predicts clinical outcome in traumatic brain injury. Sci Rep. (2016) 6:36791. 10.1038/srep36791 27819296 PMC5098187

[112] Disanto G Barro C Benkert P Naegelin Y Schädelin S Giardiello A. Serum neurofilament light: a biomarker of neuronal damage in multiple sclerosis. Ann Neurol. (2017) 81:857–70. 10.1002/ana.24954 28512753 PMC5519945

[113] Bittner S Oh J Havrdová EK Tintoré M Zipp F. The potential of serum neurofilament as biomarker for multiple sclerosis. Brain. (2021) 144:2954–63. 10.1093/brain/awab241 34180982 PMC8634125

